# Anthracycline-induced cardiotoxicity associated with myocardial energy metabolism: mechanisms revealed through an integration of ^18^F-FDG PET/CT and data-independent acquisition proteomics

**DOI:** 10.3389/fcvm.2025.1726943

**Published:** 2026-01-02

**Authors:** Yining Xu, Yihan Chen, Shujie Zhang, Biyun Zhang, Yao Zhu, Haiwen Ni, Weimin Jiang

**Affiliations:** 1 Department of Cardiology, Affiliated Hospital of Nanjing University of Chinese Medicine, Nanjing, Jiangsu, China; 2 Department of Nuclear Medicine, Affiliated Hospital of Nanjing University of Chinese Medicine, Nanjing, Jiangsu, China; 3 Department of Hematology, Affiliated Hospital of Nanjing University of Chinese Medicine, Nanjing, Jiangsu, China

**Keywords:** ^18^F-FDG PET/CT, anthracyclines, cardiotoxicity, DIA proteomics, fatty acid oxidation, glucose uptake

## Abstract

**Background and objectives:**

Anthracyclines, as a type of chemotherapy drugs, are widely used in the first-line treatment of cancers. However, anthracycline-induced cardiotoxicity (AIC) in the cumulative and dose-dependent manner greatly limits the clinical application. Insufficient energy supply caused by myocardial metabolic reconstruction is an established factor for AIC. At present, the left ventricular maximum standardized uptake value (LV SUV_max_) and left ventricular mean standardized uptake value (LV SUV_mean_) detected via ^18^F-fluorodeoxyglucose (^18^F-FDG) positron emission tomography/computed tomography (PET/CT) are effective metrics reflecting alterations of myocardial metabolism after anthracycline medications, serving as promising targets for predicting the risk of AIC. To investigate AIC-induced myocardial metabolism changes by an integration of ^18^F-FDG PET/CT and data-independent acquisition (DIA) proteomics, thus providing novel targets for predicting AIC.

**Methods:**

A total of 30 patients with diffuse large B-cell lymphomas and treated with anthracycline-based chemotherapy in the Hematology Department of Jiangsu Province Hospital of Chinese Medicine from December 2023 to December 2024 were enrolled. Finally, 17 participants were included as the Diffuse Large B-cell Lymphoma (DLBCL) group. Additionally, 13 non-oncologic participants without organic heart disease, who required ^18^F-FDG PET/CT for disease screening, were recruited as the control group. General data, dosage of anthracyclines, physical and chemical examination findings, and cardiac function indicators were collected. ^18^F-FDG PET/CT was performed before and after 6 cycles of chemotherapy in the DLBCL group, whereas the control group underwent a single scan during the same period. Serum samples were harvested for analyzing alterations of myocardial metabolism before and after chemotherapy via DIA proteomics.

**Results:**

Among 17 patients in the DLBCL group, 6 received epirubicin chemotherapy with an average dose of 59.28 ± 12.54 mg/m^2^ per cycle, and 11 received liposomal doxorubicin chemotherapy with an average dose of 25.21 ± 3.14 mg/m^2^ per cycle. Compared to the control group, no significant differences were observed in pre-chemotherapy LV SUV_max_ (median: 2.40, 95% CI: 2.04–2.74) and LV SUV_mean_ (median: 1.34, 95% CI: 1.15–1.55) in the DLBCL group (*P* > 0.05). After anthracycline-based chemotherapy, both LV SUV_max_ (median: 4.82, 95% CI: 3.50–6.05) and LV SUV_mean_ (median: 1.93, 95% CI: 1.57–2.56) significantly increased compared to pre-chemotherapy values (*P* < 0.05). The proportion of patients without ^18^F-FDG uptake in the left ventricular myocardium significantly decreased relative to both pre-chemotherapy levels and the control group (*P* < 0.001). Conversely, the proportions of patients exhibiting diffuse uptake and focal-on-diffuse uptake significantly increased compared to pre-chemotherapy measurements (*P* < 0.01). Moreover, the incidence of abnormal ^18^F-FDG uptake in the left ventricular myocardium was significantly higher after chemotherapy than both before treatment and in the control group (*P* < 0.001). DIA proteomics showed that CPT1A, ACOX1, ECH1, and ACAT1 were significantly downregulated after anthracycline-based chemotherapy, which were mainly enriched in fatty acid metabolism. Their protein levels were negatively associated with left ventricular standardized uptake value (LV SUV). The Mantel test consistently proved a significant association between fatty acid metabolism and LV SUV.

**Conclusion:**

An integration of ^18^F-FDG PET/CT and DIA proteomics reveals a decreased fatty acid oxidation (FAO) and an increased myocardial glucose uptake after anthracycline-based chemotherapy, serving as potential mechanisms of AIC. Alterations of myocardial metabolism monitored by ^18^F-FDG PET/CT may represent early indicators of metabolic remodeling, potentially identifying patients at risk for AIC.

**Clinical Trial Registration:**

https://www.chictr.org.cn/, identifier ChiCTR2400088740.

## 1 Introduction

Cardiovascular diseases (CVDs) are the second leading cause of mortality among survivors of malignant tumors (1). Routine chemotherapy and targeted therapy for cancer patients increase the risk of cardiac injury. Application of chemotherapy drugs can be challenging due to the insufficient cardiac reserve and multiple risk factors for CVDs prior to chemotherapy. Cardiac insufficiency and heart failure (HF) are the most serious cardiovascular damages in the treatment of systemic malignant tumors. Conventional chemotherapy drugs, such as anthracyclines, anti-metabolic drugs and cyclophosphamide can induce cardiomyocyte damage, resulting in acute or chronic left heart insufficiency (2). Considering the high cardiovascular risk in cancer patients, the European Society of Cardiology (ESC) recommends an alert to HF in cancer survivors. The European Society of Oncology (ESMO) also proposed that the potential negative impact of chemotherapy on cardiovascular health may offset its efficacy in treating cancer. While chemotherapy provides survival benefits, it is also important to minimize the cardiovascular risks (3). An effective, real-time, accurate monitoring of cardiotoxicity, as a result, is of profound significance during chemotherapy.

Anthracyclines like doxorubicin, epirubicin and daunorubicin are first-line drugs to treat cancers (e.g., lymphoma, sarcoma, and breast cancer) through interfering with DNA replication and transcription, inhibiting type II topoisomerases, generating excessive reactive oxygen species (ROS), and inducing cell apoptosis (4). However, the cumulative and dose-dependent anthracycline-induced cardiotoxicity (AIC) inflates the risk of adverse cardiovascular events like ischemic heart disease, HF and cardiomyopathy in cancer patients, greatly limiting the clinical application (5, 6). The incidence of CVDs within 5, 10 and 15 years is doubled in cancer patients treated with doxorubicin for a cumulative dosage of 500 mg/m^2^ and above than those treated with non-anthracycline chemotherapy (7). The risk of CVD-associated death in anthracycline-treated patients even exceeds that caused by cancer itself (8).

Electrocardiography, the cardiac enzyme test, and cardiac ultrasonography are common techniques to monitor AIC. Nevertheless, these indicators are less sensitive, and unable to predict early-stage CVDs (9, 10). A growing number of studies have laid particular emphasis on the early monitoring of AIC. ^18^F-fluorodeoxyglucose (^18^F-FDG) positron emission tomography/computed tomography (PET/CT) is an effective nuclear medical tool that is widely used in the diagnosis, staging and efficacy evaluation of various types of cancers. It also provides valuable information to assess coronary atherosclerosis, ischemic heart disease and cardiac sarcoidosis (11–14). In 2012, Borde et al. (15) for the first time proposed FDG uptake as a potential target for monitoring AIC. An abnormal uptake of ^18^F-FDG during and after anthracycline-based chemotherapy is an alerting sign of changes in myocardial metabolism and progressive increase in glucose uptake. It is also linked with the incidence of adverse cardiovascular events. Therefore, ^18^F-FDG PET/CT has certain value for predicting AIC in the early stage (16, 17).

In the present study, we followed up patients with diffuse large B-cell lymphomas and observed changes in the myocardial ^18^F-FDG uptake before and after anthracycline-based chemotherapy. Meanwhile, data-independent acquisition (DIA) proteomics was performed to analyze the influence of anthracyclines on glucose uptake and fatty acid metabolism in myocardium. Innovatively through an integration of ^18^F-FDG PET/CT and DIA proteomics, our findings provide insights into clinical early monitoring of AIC.

## 2 Methods

### 2.1 Participants

This was a prospective, observational study conducted in Jiangsu Province Hospital of Chinese Medicine between December 2023 and December 2024. Adult patients aged 18–80 years with diffuse large B-cell lymphomas (DLBCL) and treated with anthracycline-based chemotherapy were enrolled. This study was registered on the Chinese Clinical Trial Registry (https://www.chictr.org.cn/) (No. ChiCTR2400088740), and study protocols were approved by the Ethics Committee of Jiangsu Province Hospital of Chinese Medicine (No. 2023NL-240-05).

Exclusion criteria: (1) abnormal myocardial uptake on baseline ^18^F-FDG PET/CT; (2) comorbid organic cardiovascular disease; (3) diabetes mellitus, or acute conditions affecting myocardial glucose uptake such as inflammation, infection, or myocardial ischemia; (4) other primary severe diseases involving the heart, liver, lungs, kidneys, or hematopoietic system that could compromise survival (e.g., hepatocellular carcinoma, renal failure, leukemia); (5) concurrent use of other cardiotoxic drugs; (6) women in pregnancy or lactation, or individuals preparing for pregnancy; (7) allergic constitution or known hypersensitivity to ^18^F-FDG; (8) physical disability, or history of drug or alcohol abuse; (9) individuals who were unable to be cooperated due to intellectual or mental illnesses.

Additionally, a group of non-oncology patients without organic heart disease, who required ^18^F-FDG PET/CT for diagnostic screening, was also recruited. All participants provided written informed consent.

### 2.2 Study regimens

Patients with diffuse large B-cell lymphomas were treated with 6 cycles of the R-CHOP regimen, with 21 days per cycle. Briefly, the R-CHOP regimen consisted of rituximab (375 mg/m^2^ administered intravenously on day 0), cyclophosphamide (750 mg/m^2^ administered intravenously on day 1), epirubicin (70 mg/m^2^ administered intravenously on day 1) or doxorubicin liposomes (25 mg/m^2^ administered intravenously on day 1), vindesine (4 mg administered intravenously on day 1), and prednisone (60 mg/m^2^ administered intravenously) or dexamethasone (an equivalent dose administered orally on days 1–5). The cumulative dose of anthracyclines per cycle was calculated, and converted to doxorubicin-equivalent doses using established ratios (epirubicin: doxorubicin = 0.8:1; liposomal doxorubicin: doxorubicin = 1:1) to standardize the assessment of cardiotoxic exposure (1). Dosages could be adjusted based on the comprehensive assessment and individualized physical condition. Patients in the control group did not receive any drug intervention.

Baseline characteristics, including demographic data, laboratory testing data, cardiac function data, medical history, and cardiovascular risk factors, were recorded. Measurements of left ventricular maximum standardized uptake value (LV SUV_max_) and left ventricular mean standardized uptake value (LV SUV_mean_) detected via ^18^F-FDG PET/CT, and the analysis of ^18^F-FDG uptake pattern were conducted. Briefly, ^18^F-FDG PET/CT was performed once before the first session of chemotherapy, and once within 7 days of the final session in the DLBCL group. It was conducted only once during the experimental period in the control group. Serum samples were harvested for DIA proteomics.

### 2.3 ^18^F-FDG PET/CT and uptake pattern analysis

^18^F-FDG PET/CT was performed using a Siemens Biograph mCT PET/CT scanner, with qualified ^18^F-FDG provided by AMS Co., Ltd., Nanjing, China (radiochemical purity >99%, negativity for endotoxin and bacteriological testing). The primary dietary preparation was initiated at least 24 h prior to imaging and consisted of an extremely low-carbohydrate regimen (<5 g/day) with high fat and high protein content, followed by a fasting period of at least 6 h; patient compliance was ensured through standardized hospital meals and professional guidance from medical staff. Blood glucose was controlled lower than 11.1 mmol/L before the scan. Patients were administered intravenously with ^18^F-FDG at a dose of 3.7–5.5 MBq/kg, and remained in a quiet, resting state during the uptake phase. They were asked to urinate 50 min after injecting ^18^F-FDG, and drink 500 mL of water or milk to fill in the stomach. Heparin was not used before the examination, and ECG gating was not used during the examination. Weight-normalized SUVbw was adopted. In a supine position, CT scan was conducted from the skull base to the middle and upper segments of the femur, or to the level of both feet if necessary. The following acquisition parameters were adopted: 24 slices, 1.2 mm of slice thickness, 0.8 of spiral pitch, 0.5 s/rotation of speed, 3 mm of layer thickness, 2 mm of layer spacing, 120 kV of tube voltage, 90 mA of tube current. Later, three-dimensional PET scans were collected at 2.5 min/bed for 4–6 beds. PET/CT scans were finally reconstructed via attenuation correction and iterative methods.

PET/CT scans were independently processed by two senior physicians in nuclear medicine, and any disagreement was solved by the third experienced physician. The interested, three-dimensional regions in the left ventricle (LV) were manually outlined, and LV SUV_max_ and LV SUV_mean_ were measured using the Bee Viewer Pro (Supplementary Figure S1).

Myocardial ^18^F-FDG uptake pattern in the LV was classified based on the visual analysis as previously reported (18–21): (1) no uptake: ^18^F-FDG uptake in the left ventricular myocardium equal to or lower than the blood pool activity; (2) diffuse uptake: an even distribution of ^18^F-FDG throughout the left ventricular myocardium, without a focally increased uptake; (3) focal uptake: uptake was concentrated in the central part of the left ventricular myocardium than in the surrounding region; (4) focal-on-diffuse: an increased focal uptake in certain LV myocardial segments superimposed on a context of diffuse uptake. Specifically, focal uptake and focal-on-diffuse in the LV out of the basal and papillary muscle regions were determined as a condition of abnormal ^18^F-FDG uptake.

### 2.4 DIA proteomics

Total proteins were extracted using magnetic beads in lysate at 95 ℃ for 10 min, digested in trypsin at a ratio of 1:100 at 37 ℃ for 16–18 h, cleaned up using C18 columns, and concentrated in vacuum. Then, 500 ng total peptides were isolated in the Vanquish™ Neo UHPLC System, followed by data acquisition using an Orbitrap Astral mass spectrometer. Peptides were separated using EASY-Spray™ HPLC columns (150 μm × 15 cm), with the mobile phase A of 0.1% formic acid in water, and mobile phase B of 0.1% formic acid in 80% acetonitrile. Following a gradient elution program for 6.9 min, separated peptides proceeded directly into a mass spectrometry for a 7-min DIA in positive ion mode as follows: 380–980 m/z of a full first-stage scan range, 240 k of resolution, 2 m/z of isolation window, 25% of normalized collision energy, and 150–2,000 m/z of a second-stage scan range. Second-stage mass spectrometric data were searched in the dataset of uniprot_human_UP000005640, using DIA-NN (v1.9.1) with the specific parameters: Trypsin/P, 2 missed cleavage, peptide-length range of 7–30, cysteine alkylation designated as a fixed modification.

A total of 2,607 human-derived proteins were identified via mass spectrometry. Detected proteins were filtered, and more than 50% of quantitatively measured proteins within a group were retained. Then, the filtered peak area data (Filtered) and undetected data (NaN) were filled with a numerical value 0.1, thus favoring the calculation of fold change (FC). Median normalization was performed on the data of each sample. In mass spectrometry, false discovery rate (FDR) was set at <1%, and corrected by the Benjamini-Hochberg procedure. Finally, differentially expressed proteins (DEPs) before and after anthracycline-based chemotherapy, as qualified by |log_2_FC|>0.5 and *P* < 0.05, were screened.

### 2.5 Statistical analysis

Data obtained via DIA proteomics were processed and visualized using R 4.5.0. The remaining data were analyzed by SPSS 26.0. Continuous variables within a normal distribution were expressed as mean ± standard deviation (SD); otherwise, they were expressed as median (interquartile range, IQR). Categorical variables were expressed as frequency and percentage (%). Data normality was verified using Shapiro–Wilk test. The Kruskal–Wallis test was used for comparisons of the control group and the DLBCL group before and after chemotherapy. For significant results, Dunn's *post hoc* test with Bonferroni correction was used for *post hoc* multiple comparisons. Frequency and percentage between groups were compared by Fisher's exact test. The confidence intervals for median values were calculated using the Hodges–Lehmann estimator. Spearman correlation test was used to assess the association between two samples, while Mantel test and Canonical Correlation Analysis (CCA) were applied to evaluate the relationships between matrices. The Mantel test was used to analyze the association between enriched terms or pathways and clinical indicators. CCA can effectively facilitate data integration and further validate the associations between differentially expressed proteins and clinical indicators. *P* < 0.05 was considered statistically significant.

## 3 Results

### 3.1 Baseline characteristics

A total of 30 patients with diffuse large B-cell lymphomas and treated with anthracycline-based chemotherapy were initially enrolled. Among them, 11 were excluded due to abnormal myocardial uptake on baseline ^18^F-FDG PET/CT, and 2 were excluded due to comorbid diabetes mellitus. Consequently, 17 eligible participants were included as the Diffuse Large B-cell Lymphoma (DLBCL) group. Additionally, 13 other patients were recruited as the control group. The cumulative dose per cycle for epirubicin was 59.28 ± 12.54 mg/m^2^, and for liposomal doxorubicin, it was 25.21 ± 3.14 mg/m^2^. The total cumulative doxorubicin-equivalent dose for epirubicin was 284.54 ± 60.20 mg/m^2^, and for liposomal doxorubicin, it was 151.27 ± 18.80 mg/m^2^. To evaluate possible selection biases, we characterized excluded participants, showing comparable baseline characteristics, including age, gender, height, weight and body mass index (BMI) with eligible DLBCL patients (Supplementary Table S1).

The DLBCL group had a mean age of 63.65 ± 9.91 years, consisting of 11 males and 6 females. Among these patients, 6 were treated with epirubicin and 11 received liposomal doxorubicin. The control group had a mean age of 58.15 ± 6.69 years, comprising 5 males and 8 females. Detailed baseline characteristics of both groups were listed in Table 1.

**Table 1:** Baseline characteristics of participants.

Variables	DLBCL (*n* = 17)	Control (*n* = 13)	*P*-value
Characteristic			
Age (years)	63.65 ± 9.91	58.15 ± 6.69	0.187
Male sex (*n*, %)	11 (64.7)	5 (38.5)	0.269
Height (cm)	166.88 ± 10.24	168.23 ± 8.47	0.704
Weight (kg)	61.76 ± 15.08	67.00 ± 12.97	0.326
BMI (kg/m^2^)	22.01 ± 3.93	23.49 ± 2.84	0.261
Anthracyclines (*n*, %)			
Epirubicin	6 (35.3)	–	–
Doxorubicin liposomes	11 (64.7)	–	–
Cumulative doses per cycle (mg/m^2^)			
Epirubicin	59.28 ± 12.54	–	–
Doxorubicin liposomes	25.21 ± 3.14	–	–
Cumulative doxorubicin-equivalent doses (mg/m^2^)			
Epirubicin	284.54 ± 60.20	–	–
Doxorubicin liposomes	151.27 ± 18.80	–	–
Hypertension (*n*, %)	6 (35.3)	3 (23.1)	0.691
Hyperlipidemia (*n*, %)	3 (17.6)	2 (15.4)	1.000
Smoking (*n*, %)	3 (17.6)	1 (7.7)	0.613
Alcohol consumption (*n*, %)	1 (5.9)	3 (23.1)	0.290

DLBCL, diffuse large B-cell lymphomas; BMI, body mass index.

### 3.2 Clinical data and echocardiogram findings

Compared with the control group, the DLBCL group exhibited significantly lower lymphocyte counts (LYMPH#) both before and after chemotherapy (*P* < 0.05, *P* < 0.001), while monocyte counts (MONO#) were significantly elevated after chemotherapy (*P* < 0.01). Additionally, before chemotherapy, creatine kinase (CK) was significantly lower (*P* < 0.05), whereas lactate dehydrogenase (LDH) was significantly higher (*P* < 0.01) in the DLBCL group compared with controls. However, no significant differences were observed in these markers within the DLBCL group before vs. after chemotherapy (*P* > 0.05).

Other biochemical parameters, including creatine kinase-MB (CK-MB), uric acid (UA), low-density lipoprotein (LDL), and NT-proBNP, showed no significant differences between the control group and the DLBCL group at either baseline or post-chemotherapy time points (*P* > 0.05). Echocardiographic parameters such as left atrial diameter (LAD), left ventricular internal dimension at end-diastole (LVIDd), interventricular septal diameter (IVSd), left ventricular ejection fraction (LVEF), and E/e′ (the ratio of peak early mitral inflow velocity to the early diastolic mitral annular velocity) also demonstrated no significant differences in inter-group comparisons at either time point (*P* > 0.05) (Table 2).

**Table 2:** Clinical data and echocardiogram findings before and after anthracycline-based chemotherapy.

Variables	Control (*n* = 13)	Before (*n* = 17)	After (*n* = 17)	*P*-value
Clinical data				
WBC (10^9^/L)	5.82 (4.65, 6.57)	5.42 (3.39, 6.69)	4.58 (4.30, 5.10)	0.248
NEUT# (10^9^/L)	3.23 (2.54, 4.22)	3.37 (2.00, 4.22)	2.57 (1.97, 3.52)	0.414
LYMPH# (10^9^/L)	1.97 (1.64, 2.28)	1.07 (0.80, 1.34)*	0.88 (0.62, 1.07)***	0.001
MONO# (10^9^/L)	0.42 (0.36, 0.49)	0.50 (0.34, 0.77)	0.63 (0.52, 0.74)**	0.010
CK (U/L)	75.00 (51.50, 93.00)	45.00 (29.50, 65.75)*	49.50 (37.75, 64.50)	0.014
CK-MB (U/L)	12.00 (9.00, 18.00)	13.50 (11.00, 16.50)	12.00 (10.00, 15.50)	0.733
LDH (U/L)	185.00 (161.00, 210.50)	245.00 (199.00, 367.00)**	217.00 (184.00, 254.75)	0.006
AST (U/L)	19.00 (16.00, 24.50)	19.00 (17.00, 23.50)	22.00 (18.00, 24.00)	0.587
UA (μmol/L)	290.00 (263.00, 370.00)	312.00 (246.00, 358.00)	342 (290.00, 377.00)	0.205
TC (mmol/L)	4.11 ± 0.74	4.36 ± 0.96	4.56 ± 0.87	0.396
TG (mmol/L)	1.06 (0.81, 1.58)	1.14 (0.93, 1.82)	1.38 (1.01, 1.89)	0.365
HDL (mmol/L)	1.41 ± 0.33	1.26 ± 0.43	1.22 ± 0.23	0.328
LDL (mmol/L)	2.03 (1.61, 2.93)	2.34 (2.07, 2.64)	2.27 (1.90, 2.90)	0.731
NT-proBNP (pg/ml)	3.41 (1.62, 7.09)	7.55 (3.02, 18.30)	7.75 (1.73, 13.75)	0.243
Echocardiogram				
LAD (mm)	31.46 ± 5.58	33.92 ± 4.58	36.29 ± 5.33	0.068
LVIDd (mm)	45.38 ± 3.33	46.08 ± 5.50	48.21 ± 4.59	0.252
LVIDs (mm)	28.38 ± 3.15	29.25 ± 3.47	30.93 ± 3.34	0.142
IVSd (mm)	10.00 (8.00, 10.00)	9.00 (9.00, 10.38)	10.00 (9.00, 10.50)	0.478
LVPWd (mm)	9.00 (8.00, 10.00)	9.00 (8.63, 9.00)	9.00 (9.00, 9.50)	0.883
LVEF (%)	67.66 ± 3.88	66.23 ± 2.83	65.28 ± 3.59	0.217
E/e’	8.28 ± 2.19	9.31 ± 2.10	8.55 ± 2.04	0.458

WBC, white blood cell count; NEUT#, neutrophil count; LYMPH#, lymphocyte count; MONO#, monocyte count; CK, creatine kinase; CK-MB, creatine kinase-MB; LDH, lactate dehydrogenase; AST, aspartate aminotransferase; UA, uric acid; TC, total cholesterol; TG, triglycerides; HDL, high-density lipoprotein; LDL, low-density lipoprotein; NT-proBNP, N-terminal pro-B-type natriuretic peptide; LAD, left atrial diameter; LVIDd, left ventricular internal dimension at end-diastole; LVIDs, left ventricular internal dimension at end-systole; IVSd, interventricular septal diameter; LVPWd, eft ventricular posterior wall thickness at end-diastole; LVEF, left ventricular ejection fraction; E/e’, the ratio of peak early mitral inflow velocity (E) to the early diastolic mitral annular velocity (e’). Compared with the control group.

* *P* < 0.05.

** *P* < 0.01.

*** *P* < 0.001.

### 3.3 ^18^F-FDG PET/CT myocardial metabolism

Myocardial metabolic parameters measured by ^18^F-FDG PET/CT were compared between the control group and the DLBCL group before and after the latter received six cycles of anthracycline-based chemotherapy (Table 3, Supplementary Table S2). Before chemotherapy, no significant differences were observed in LV SUV_max_ (median: 2.45 vs. 2.40; 95% CI: 2.27–3.68 vs.2.04–2.74) or LV SUV_mean_ (median: 1.64 vs. 1.34; 95% CI: 1.51–2.03 vs.1.15–1.55) between the two groups (both *P* > 0.05). After chemotherapy, LV SUV_max_ significantly increased to 4.82 (95% CI, 3.50–6.05) compared to both the control group (*P* < 0.01) and the pre-chemotherapy baseline (*P* < 0.001). Similarly, LV SUV_mean_ increased to 1.93 (95% CI, 1.57–2.56), which was significantly higher than the pre-chemotherapy level (*P* < 0.001) but not significantly different from the control group (*P* > 0.05).

**Table 3:** Myocardial metabolism parameters before and after anthracycline-based chemotherapy.

Variables	Control (*n* = 13)		Before (*n* = 17)		After (*n* = 17)		*P*-value
	M (IQR)	95% CI	M (IQR)	95% CI	M (IQR)	95% CI	
LV SUV_max_	2.45 (2.13, 3.63)	2.27–3.68	2.40 (1.88, 2.66)	2.04–2.74	4.82 (3.18, 6.95)**###	3.50–6.05	<0.001
LV SUV_mean_	1.64 (1.41, 2.12)	1.51–2.03	1.34 (1.14, 1.58)	1.15–1.55	1.93 (1.65, 2.92)###	1.57–2.56	<0.001

LV SUV_max_, left ventricular maximum standardized uptake value; LV SUV_mean_, left ventricular mean standardized uptake value. Compared with the control group, **P < 0.01; compared with pre-chemotherapy, ###*P* < 0.001.

**Figure 1:**
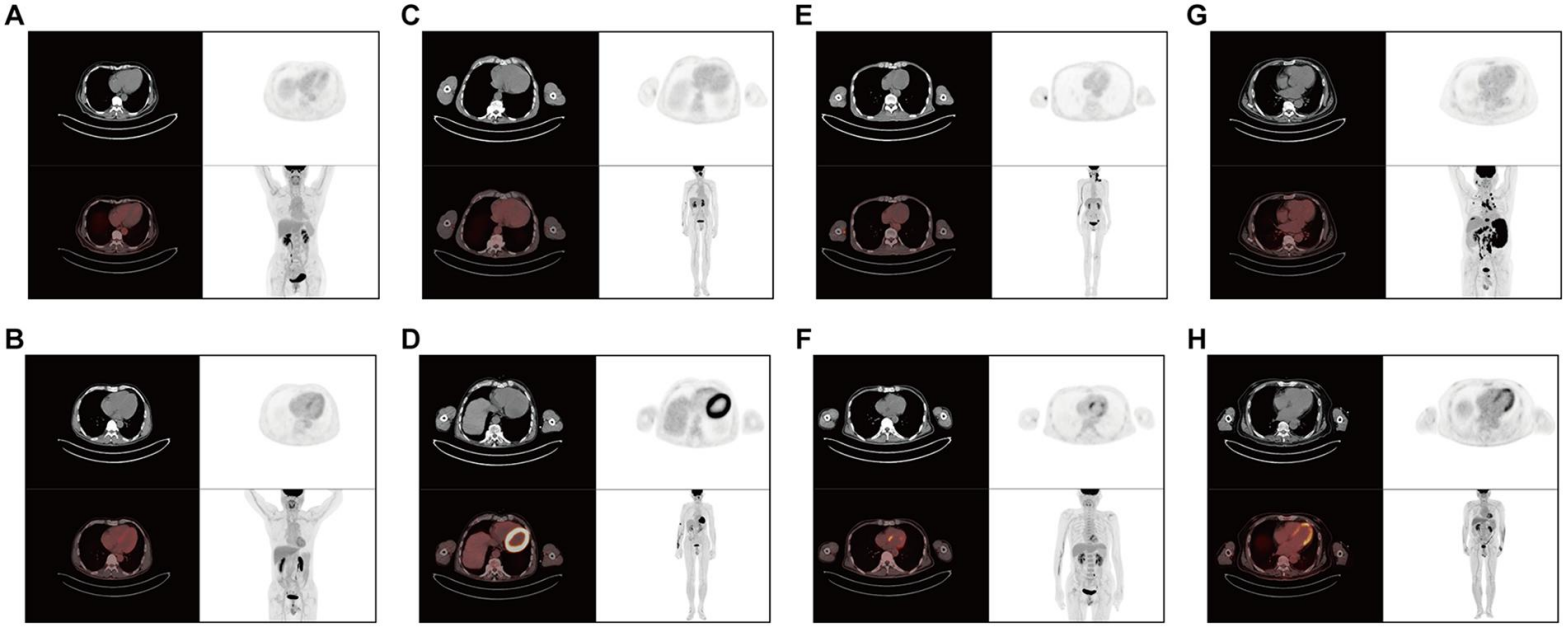
Representative PET/CT scans of myocardial ^18^F-FDG uptake pattern in the left ventricle. **(A,B)** A patient from the control group showing no myocardial 18F-FDG uptake **(A)**, and another patient from the control group demonstrating mild diffuse myocardial uptake **(B)**. **(C,D)** A patient with pre-chemotherapy no myocardial ^18^F-FDG uptake **(C)**, but a diffuse, circular uptake after 6 cycles of R-CHOP **(C)**. **(E,F)** A patient with pre-chemotherapy no myocardial ^18^F-FDG uptake **(E)**, but a focal uptake after 6 cycles of R-CHOP **(F)**. **(G,H)** A patient with pre-chemotherapy no myocardial ^18^F-FDG uptake **(G)**, but a focal-on-diffuse pattern (focal uptake in the apex) after 6 cycles of R-CHOP **(H)**.

In addition, the myocardial ^18^F-FDG uptake pattern was analyzed (Table 4, Figure 1). The number of cases with no left ventricular uptake decreased significantly in the DLBCL group after 6 cycles of chemotherapy compared to both the pre-chemotherapy assessment and the control group (*P* < 0.001). Furthermore, the incidences of diffuse uptake and focal-on-diffuse uptake significantly increased post-chemotherapy compared to pre-chemotherapy values (*P* < 0.01). However, the number of cases exhibiting focal uptake did not differ significantly among the control group or within the DLBCL group before and after treatment (*P* > 0.05). Additionally, the prevalence of abnormal left ventricular FDG uptake was significantly higher after chemotherapy than both at baseline and in the control group (*P* < 0.001).

**Table 4:** Myocardial ^18^F-FDG uptake pattern on PET/CT scans before and after anthracycline-based chemotherapy.

Uptake pattern	Control (*n* = 13)	Before (*n* = 17)	After (*n* = 17)	*P*-value
No uptake (*n*, %)	9 (69.2)	16 (94.1)	0 (0.0)***###	<0.001
Diffuse uptake (*n*, %)	4 (30.8)	1 (5.9)	9 (52.9)##	0.008
Focal uptake (*n*, %)	0 (0.0)	0 (0.0)	3 (17.6)	0.102
Focal-on-diffuse (*n*, %)	0 (0.0)	0 (0.0)	5 (29.4)##	0.009
Abnormal uptake (*n*, %)	0 (0.0)	0 (0.0)	8 (41.1)***###	<0.001

18 F-FDG, ^18^F-fluorodeoxyglucose; PET, positron emission tomography; CT, computed tomography. Compared with the control group, ***P < 0.001; compared with pre-chemotherapy, ##*P* < 0.01, ###*P* < 0.001.

### 3.4 DEPs before and after anthracycline-based chemotherapy

**Figure 2:**
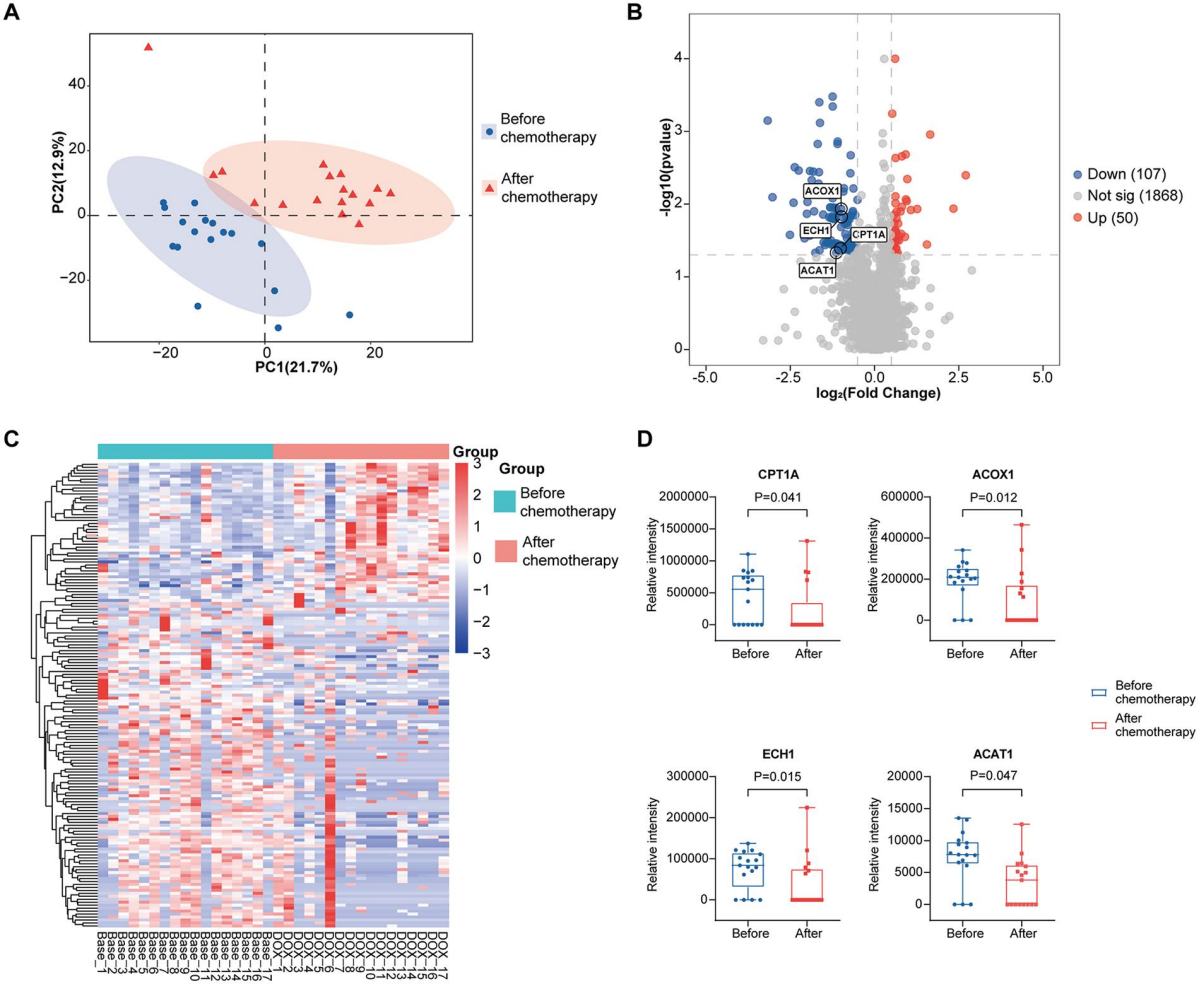
Changes in DIA proteomics before and after anthracycline-based chemotherapy. **(A)** PLS-DA classifies protein distribution before and after anthracycline-based chemotherapy. **(B,C)** Volcano plots **(B)** and heatmap **(C)** visualizing differentially expressed proteins before and after anthracycline-based chemotherapy. **(D)** Quantitative analyses of relative levels of CPT1A, ACOX1, ECH1 and ACAT1. *P < 0.05 vs. before chemotherapy.

To further investigate the potential mechanism by which anthracycline drugs disrupt myocardial metabolism and induce cardiac injury, we performed data-independent acquisition (DIA) proteomic analysis on serum samples collected from patients in the DLBCL group before and after chemotherapy. Protein distribution was distinctly different in patients with diffuse large B-cell lymphomas before and after 6 cycles of anthracycline-based chemotherapy (Figure 2A). DIA identified 157 DEPs (|log_2_ fold change|<0.5, *P* < 0.05), involving 50 upregulated, and 107 downregulated proteins (Figure 2B). Expression levels of individual DEPs were visualized by the clustering analysis (Figure 2C). In addition, DEPs were annotated in the UniProt. A box of key proteins associated with fatty acid metabolism was found significantly downregulated after anthracycline-based chemotherapy, including CPT1A, ACOX1, ECH1 and ACAT1 (*P* < 0.05) (Figure 2D).

### 3.5 GO and KEGG pathway analyses of DEPs

**Figure 3:**
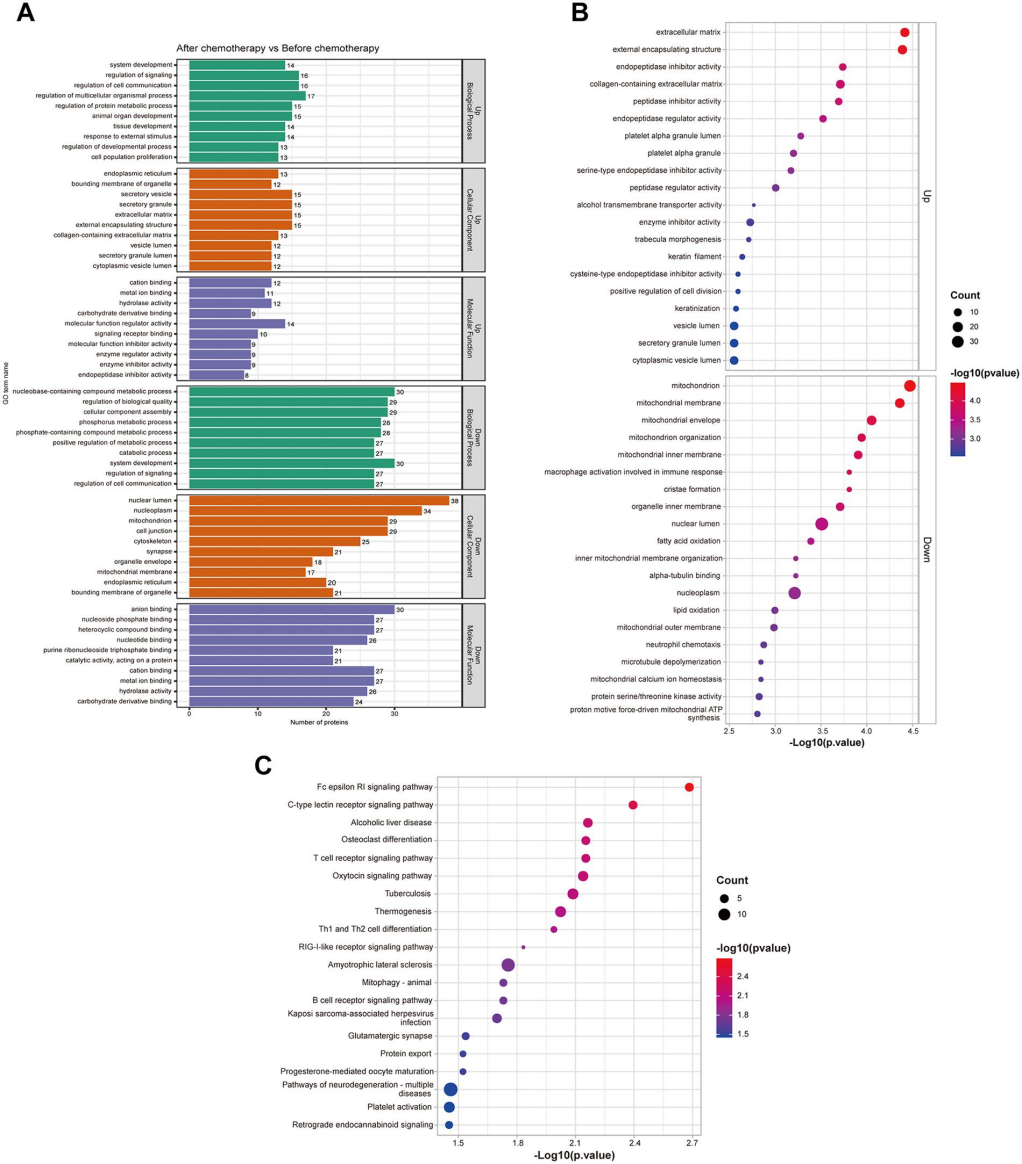
GO and KEGG pathway analyses of DEPs before and after anthracycline-based chemotherapy. **(A)** GO term annotations of DEPs. **(B)** The top 20 upregulated and downregulated GO terms. **(C)** The top 20 enriched signaling pathways predicted by KEGG pathway analysis.

Biological process (BP), cellular component (CC) and molecular function (MF) of DEPs were annotated. It is found that DEPs were mainly enriched in the regulation of signaling, endoplasmic reticulum and cation binding (Figure 3A). Furthermore, a significant downregulation of fatty acid oxidation (FAO) was predicted, and GO terms associated with mitochondrial structure and function were also associated with DEPs (Figure 3B). KEGG pathway analysis revealed the enrichment of signaling pathways associated with metabolism and energy regulation, such as thermogenesis and mitophagy-animal (Figure 3C).

### 3.6 Associations of DEPs with cardiac metabolism

**Figure 4:**
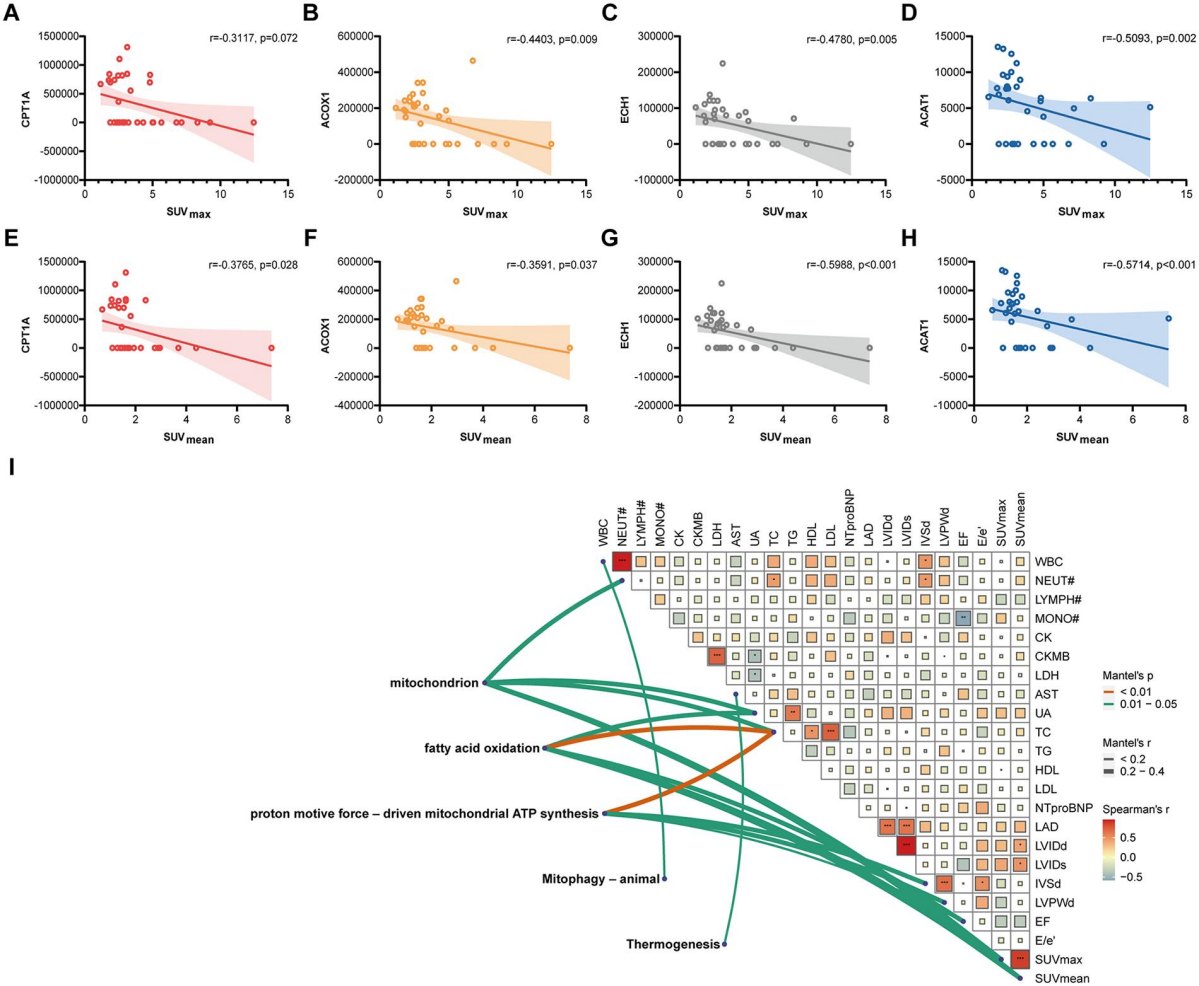
Associations of DEPs with cardiac metabolism. **(A)** Association of CPT1A with LV SUV_max_ (*r* = −0.3117, *P* = 0.072). **(B)** Association of ACOX1 with LV SUV_max_ (*r* = −0.4403, *P* = 0.009). **(C)** Association of ECH1 with LV SUV_max_ (*r* = −0.4780, *P* = 0.005). **(D)** Association of ACAT1 with LV SUV_max_ (*r* = −0.5093, *P* = 0.002). **(E)** Association of CPT1A with LV SUV_mean_ (*r* = −0.3765, *P* = 0.028). **(F)** Association of ACOX1 with LV SUV_mean_ (*r* = −0.3591, *P* = 0.037). **(G)** Association of ECH1 with LV SUV_mean_ (*r* = −0.5988, *P* < 0.001). **(H)** Association of ACAT1 with LV SUV_mean_ (*r* = −0.5714, *P* < 0.001). **(I)** The Mantel test visualizing correlations of GO terms and KEGG pathways enriched in DEPs with clinical data and myocardial metabolism parameters.

**Figure 5:**
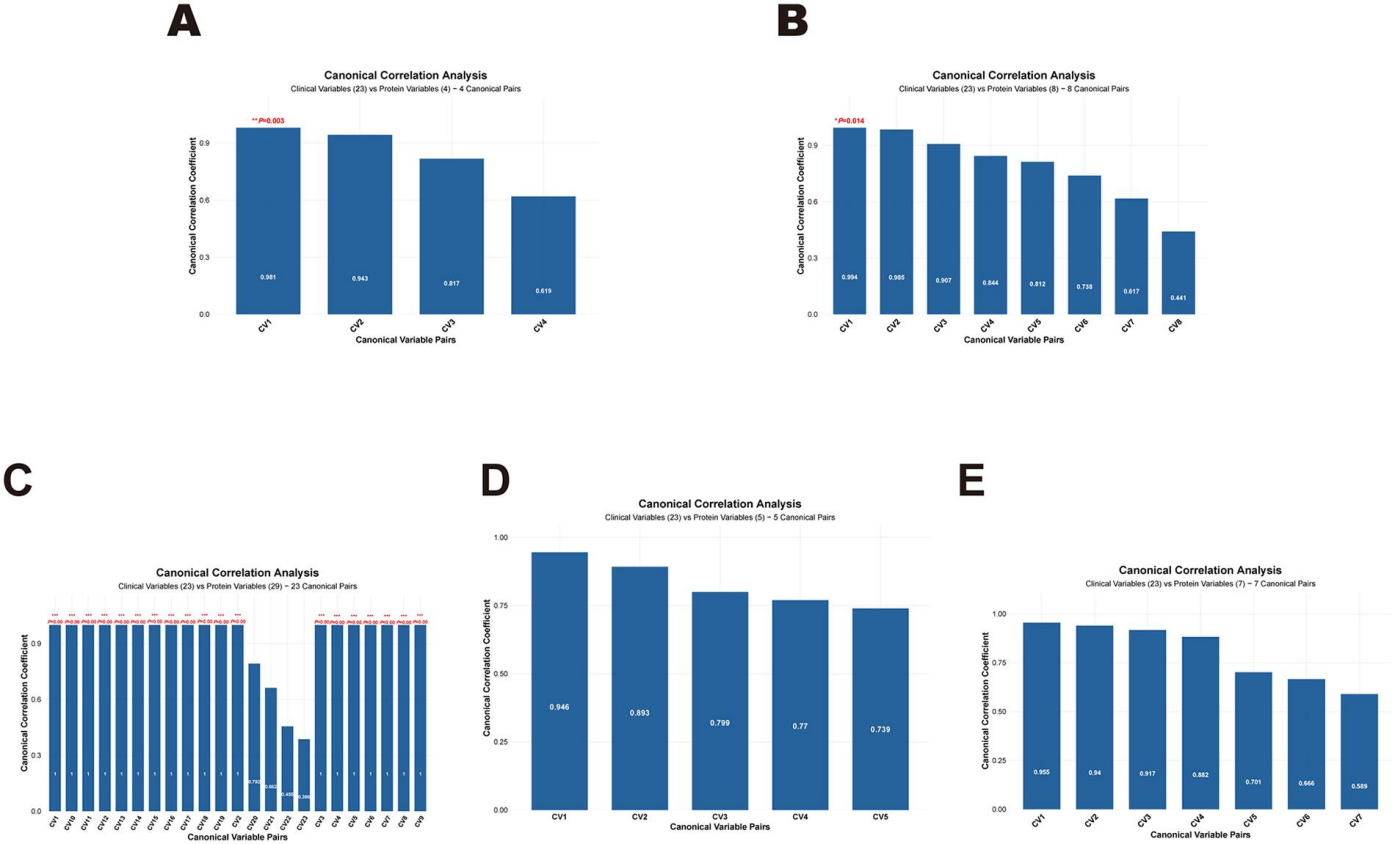
Bar plots of canonical correlation coefficients for DEPs associated with functional enrichment terms and myocardial metabolic parameters based on CCA. **(A)** Mitophagy-animal. **(B)** Thermogenesis. **(C)** Mitochondrion. **(D)** Proton motive force-driven mitochondrial ATP synthesis. **(E)** Fatty acid oxidation. *P < 0.05, **P < 0.01, ***P < 0.001.

We analyzed associations of CPT1A, ACOX1, ECH1 and ACAT1 with LV myocardial ^18^F-FDG uptake. While CPT1A showed a negative association with LV SUV_max_, a significant difference was not detected (*P* > 0.05) (Figure 4A). ACOX1, ECH1 and ACAT1 were all significantly associated with LV SUV_max_ in a negative mode (all *P* < 0.05) (Figures 4B–D). In addition, CPT1A, ACOX1, ECH1 and ACAT1 were negatively associated with LV SUV_mean_ (all *P* < 0.05) (Figures 4E–H). The Mantel test assessed the associations of GO terms and KEGG pathways enriched in DEPs with clinical data and myocardial metabolism parameters. It is found that “Fatty Acid Oxidation” and “Mitochondrion” were significantly associated with LV SUV_max_ and LV SUV_mean_ (*P* < 0.05). Meanwhile, “Fatty Acid Oxidation” was also associated with LVEF (*P* < 0.05) (Figure 4I). Subsequently, we performed CCA to further validate the results of the Mantel test (Figure 5). The results revealed that among the canonical variable pairs associated with “Mitophagy-animal”, “Thermogenesis”, and “Mitochondrion”-related DEPs, respectively, there were significant correlations with canonical correlation coefficients greater than 0.9 (*P* < 0.05, Figures 5A–C). Specifically, in the first canonical variable pair (CV1) constructed by DEPs related to “Mitophagy-animal” (Figure 5A), the top five clinical variables by loadings were LAD, SUV_mean_, AST, SUV_max_, and LVPWd (Supplementary Table S3). In the canonical variable pair corresponding to DEPs related to “Thermogenesis” (CV1, Figure 5B), the top five clinical variables by loadings were EF, SUV_max_, SUV_mean_, CK, and E/e’ (Supplementary Table S4). Furthermore, DEPs related to “Mitochondrion” were involved in a total of 19 canonical variable pairs (Figure 5C), and SUV_max_ and SUV_mean_ were consistently included among the top five clinical variables by loadings across these pairs (Supplementary Table S5).

## 4 Discussion

The exact mechanism of AIC has not been fully elucidated. Previous evidence confirms that excess ROS and type II topoisomerase-induced DNA damage, oxidative stress, cell apoptosis and autophagy are established factors influencing AIC (22, 23). Latest studies show that anthracyclines play a key role in cardiac injuries by interfering with myocardial energy metabolism. Under physiological conditions, adenosine triphosphate (ATP) is produced by various substrates (e.g., carbohydrates, lipids, lactic acid, amino acids, ketone bodies) in the heart of healthy adults to supply energy, among which fatty acid β oxidation is the predominant pathway for energy metabolism (24). Medication with anthracyclines transforms the high-efficacy energy production in cardiomyocytes via FAO to the low-efficacy mode via glycolysis, leading to an insufficient supply of myocardial energy. Consequently, myocardial contraction and diastolic dysfunction ultimately induce various cardiovascular complications (25–27).

In this study, we first inhibited the physiological uptake of glucose by cardiomyocytes through dietary control. Under this premise, LV SUV_max_ and LV SUV_mean_ on ^18^F-FDG PET/CT scans significantly increased after anthracycline-based chemotherapy, suggesting the increased transfer of myocardial substrates and glucose uptake. The majority of DLBCL patients experienced an increase in SUV_max_ by 30% after chemotherapy, indicating the myocardial metabolic reconstruction (28). Moreover, the increased myocardial SUV was also associated with the decreased LVEF on echocardiography, serving as an early predictor for AIC (17). The visual analysis of myocardial ^18^F-FDG uptake pattern showed a significantly decreased proportion of no ^18^F-FDG uptake and an increased proportion of abnormal uptake. Generally, an abnormal myocardial uptake pattern, especially focal uptake, was a strong predictive factor for adverse cardiovascular events (29). It is found that patients with abnormal myocardial uptake after chemotherapy usually had a greater left atrium diameter and a lower LVEF compared with individuals presenting normal patterns, suggesting that abnormal myocardial ^18^F-FDG uptake was an alert sign of early cardiac injuries (21).

In this study, no significant differences were found in myocardial injury markers (CK and CK-MB) and cardiac function indicators (NT-proBNP, LVEF and E/e') before and after chemotherapy, which may be attributed to a small sample size, a short period of follow-up and low dosages of anthracyclines. Our findings also hinted that ^18^F-FDG PET/CT was a useful tool to reflect subclinical cardiac alterations earlier and more sensitively than conventional examinations for AIC. To further dig out the impact of anthracyclines on the myocardial energy metabolism in the early stage of chemotherapy, serum samples were harvested for DIA proteomics. A total of 157 DEPs were identified before and after anthracycline-based chemotherapy. Among them, CPT1A, ACOX1, ECH1 and ACAT1, as vital proteins positively expressed on different phases of fatty acid β-oxidation, were found significantly downregulated after chemotherapy. CPT1 is a rate-limiting enzyme for transporting long-chain fatty acids into mitochondria. Its downregulation hinders the β oxidation of fatty acids and weighs towards glucose supply in myocardium, resulting in lipid accumulation and myocardial toxicity (30). Although CPT1B is the main subtype of myocardial FAO, its downregulation after anthracycline-based chemotherapy reflected the overall changes in cardiac metabolism. Both CTP1A and CPT1B can be downregulated by anthracyclines, thus influencing energy metabolism in the heart hand by hand (31, 32). ACOX1 is the starting enzyme for β-oxidation of ultra-long chain fatty acids in peroxisomes. Previous studies suggest that knockdown of ACOX1 aggravates the insufficient energy supply of myocardium, posing a negative impact on cardiac function (33). ECH1 and ACAT1, as enzymes involved in the distal steps of fatty acid β-oxidation, reduce the production of acetyl-CoA, and ultimately lead to insufficient ATP synthesis. Collectively, downregulated CPT1A, ACOX1, ECH1 and ACAT1 synergistically impair the myocardial fatty acid metabolism pathway. Meanwhile, GO and KEGG enrichment analyses of DEPs also revealed significant enrichment of terms and pathways related to FAO and energy metabolism. These findings further indicated the inhibition of fatty acid metabolism and the reduction in energy supply following anthracycline-based chemotherapy.

Spearman's correlation analysis demonstrated that DEPs associated with FAO and mitochondrion were associated with LV SUV. It suggested that anthracyclines imbalanced the uptake and utilization of myocardial substrates. Inhibition of FAO may be compensated by increasing the utilization of glucose, although such metabolic reconstructions do not provide sufficient energy required for cardiac physiological activities. As a result, a failure of myocardial energy supply eventually triggers AIC (34). The Mantel test further elevated the analysis from single proteins to the functional pathway level. Results showed that both the “Fatty Acid Oxidation” pathway and “Mitochondrion”-related GO terms were significantly associated with SUV parameters of left ventricles. This suggests a systematic downregulation of the FAO mechanism—rather than changes in individual proteins—is a core feature of metabolic remodeling. As the primary site for FAO and oxidative phosphorylation, mitochondria naturally become the key hub in this metabolic shift; impairment of their function or biogenesis is closely related to altered myocardial substrate preference. More clinically significant is the finding that the “Fatty Acid Oxidation” pathway was significantly associated with LVEF, suggesting that decreased FAO capacity may be directly related to the worsening of myocardial contractile function and the progression of heart failure. To further dissect the complex relationships among multiple variables, we employed CCA, which validated the above findings at a more refined level: Variable pairs involving DEPs related to “Mitophagy”, “Thermogenesis”, and “Mitochondrion” all exhibited extremely high canonical correlation coefficients, indicating strong associations between these mitochondrial-related processes and specific sets of clinical metabolic parameters. Notably, across multiple significant canonical variable pairs, SUV_max_ and SUV_mean_ consistently emerged as core clinical variables, closely linked to mitophagy, thermogenesis, and mitochondrial DEPs, highlighting the central role of myocardial glucose metabolism as an indicator reflecting mitochondrial functional status.

Based on the above findings, a systematic connection between the proteome and imaging results can be proposed (Supplementary Figure S2): Anthracycline-induced cardiotoxicity triggers coordinated downregulation of key mitochondrial proteins. This involves not only core enzymes of FAO but also proteins maintaining mitochondrial quality control (e.g., those involved in mitophagy). The decreased expression of key FAO enzymes directly weakens the heart's primary energy source, leading to energy shortage, and the accumulation of fatty acid intermediates inducing lipotoxicity, which further damages mitochondrial function. Concurrently, the downregulation of mitophagy-related proteins impedes the clearance of damaged mitochondria. Faced with severe FAO impairment and mitochondrial dysfunction, cardiomyocytes initiate compensatory metabolic mechanisms, enhancing glycolysis to maintain ATP supply. Therefore, the increased ^18^F-FDG uptake observed on PET/CT is not merely a reflection of increased glucose utilization but also a functional “compensatory signal” emitted by cardiomyocytes during an energy crisis. These speculations suggest that PET/CT imaging can serve as a window for observing cellular metabolic reprogramming, thereby directly linking clinical imaging findings with the multidimensional proteomic signatures of mitochondrial dysfunction.

## 5 Strengths and limitations

The present study focused on subclinical changes in myocardial energy metabolism during the early stage of AIC. ^18^F-FDG PET/CT, as a common examination for diagnosing cancers and assessing the efficacy of anti-cancer treatment, does not additionally increase radiation exposure, examination time and medical cost. Moreover, it provides useful information to monitor an early AIC after chemotherapy, laying a clinical significance in preventing cardiovascular events among DLBCL patients. We innovatively integrated the analysis of myocardial ^18^F-FDG uptake on PET/CT scans and DIA proteomics to unmask the underlying mechanisms of AIC associated with fatty acid metabolism and glucose uptake.

Limitations existed in our study. First of all, we strictly enrolled DLBCL patients who were examined by both ^18^F-FDG PET/CT and DIA proteomics. Due to the low incidence of DLBCL in clinical settings, and the dual-examinations, a small sample size may influence the credibility of our conclusion. Second, great efforts have been made on minimizing factors associated with cardiac metabolism in non-oncogenic controls of the control group. In fact, the optimal controls are DLBCL patients without the treatment of anthracycline-based chemotherapy, who can barely be allocated due to the first-line treatment of anthracyclines. Third, ^18^F-FDG PET/CT was only performed once for disease screening in non-oncogenic individuals of the control group due to the potential radiation exposure and ethical requirements. We therefore failed to compare ^18^F-FDG PET/CT parameters between groups at the same time points. Fourth, we did not see significant differences in conventional cardiac function markers before and after anthracycline-based chemotherapy. It is also important to note that global longitudinal strain (GLS), a more sensitive and standard echocardiography-based marker for early cardiotoxicity, was not assessed in this study. A prolonged follow-up is essential in the future to monitor their changes and cardiovascular events, thus illustrating the value of ^18^F-FDG PET/CT in the early prediction of AIC. Finally, obtaining myocardial tissue for proteomic analysis involves an invasive biopsy procedure, which was precluded in this study due to ethical considerations. Consequently, we utilized serum samples as a clinically feasible alternative. The serum proteomics data were derived from clinically collected real-world samples, in which the alterations in serum proteins reflect short-term changes in the serum proteome following chemotherapy. These findings may offer valuable references for longer-term follow-up cohorts of patients with AIC. Specifically, the differentially expressed proteins identified before and after chemotherapy could serve as potential predictive candidates for subsequent cardiovascular events during extended follow-up. However, serum protein changes cannot be assumed to reflect cardiomyocyte expression. The heart resides within a complex microenvironment, and the circulating proteome integrates contributions from multiple tissues, including the myocardium, vascular endothelium, immune cells, and even distant organs. Therefore, the protein signatures we identified may originate from various sources beyond cardiomyocytes. Future validation in large-scale cohorts is essential. Moreover, the cardiac specificity of these candidate proteins should be further interrogated through direct analysis of myocardial tissues or cardiac-derived exosomes.

## 6 Conclusion

An integration of ^18^F-FDG PET/CT and DIA proteomics reveals a decreased FAO and an increased myocardial glucose uptake after anthracycline-based chemotherapy, serving as potential mechanisms of AIC. Alterations of myocardial metabolism monitored by ^18^F-FDG PET/CT may reflect early metabolic alterations associated with AIC risk.

## Data Availability

The datasets presented in this study can be found in online repositories. The names of the repository/repositories and accession number(s) can be found in the article/Supplementary Material.
