# Supplementary material for: Anthracycline-induced cardiotoxicity associated with myocardial energy metabolism: mechanisms revealed through an integration of 18F-FDG PET/CT and data-independent acquisition proteomics
Source: Front Cardiovasc Med. 2026 Jan 2;12:1726943. doi: 10.3389/fcvm.2025.1726943 (PMC12808406; doi:10.3389/fcvm.2025.1726943)
Supplement: Supplementary file 1 [file Supplementaryfile1.docx]

Supplementary Table 1. Baseline characteristics of participants.

|  | Included participants  (n=17) | Excluded participants  (n=13) | *P-value* |
| --- | --- | --- | --- |
| Age (years) | 63.65±9.91 | 59.08±6.29 | 0.158 |
| Male gender (n, %) | 11 (64.7) | 5 (38.5) | 0.269 |
| Height (cm) | 166.88±10.24 | 162.92±7.85 | 0.257 |
| Weight (kg) | 61.76±15.08 | 62.38±10.79 | 0.901 |
| BMI (kg/m^2^) | 22.01±3.93 | 23.34±2.46 | 0.293 |
| LV SUV_max_ | 2.40 (1.88, 2.66) | 3.71 (2.14, 6.88) | 0.070 |
| LV SUV_mean_ | 1.34 (1.14, 1.58) | 1.30 (1.26, 2.00) | 0.589 |

BMI, body mass index; LV SUV_max_, left ventricular maximum standardized uptake value; LV SUV_mean_, left ventricular mean standardized uptake value.

Supplementary Table 2. The raw data of SUV-related parameters.

| Sample | SUVmax | SUVmean |
| --- | --- | --- |
| Control | 2.130 | 1.289 |
| Control | 2.046 | 1.429 |
| Control | 2.545 | 1.655 |
| Control | 2.120 | 1.398 |
| Control | 2.336 | 1.584 |
| Control | 5.128 | 2.288 |
| Control | 2.453 | 1.543 |
| Control | 2.842 | 1.852 |
| Control | 1.993 | 1.356 |
| Control | 5.466 | 2.715 |
| Control | 2.394 | 1.637 |
| Control | 3.315 | 1.961 |
| Control | 3.949 | 2.316 |
| Before | 1.882 | 1.089 |
| Before | 2.538 | 1.423 |
| Before | 1.805 | 1.055 |
| Before | 2.188 | 1.184 |
| Before | 2.488 | 1.464 |
| Before | 2.455 | 1.338 |
| Before | 2.399 | 1.462 |
| Before | 2.172 | 1.280 |
| Before | 1.160 | 0.686 |
| Before | 3.132 | 1.624 |
| Before | 2.875 | 1.935 |
| Before | 2.563 | 1.200 |
| Before | 3.376 | 1.810 |
| Before | 1.763 | 1.023 |
| Before | 2.747 | 1.579 |
| Before | 1.883 | 1.341 |
| Before | 2.378 | 1.585 |
| After | 2.758 | 1.615 |
| After | 4.821 | 2.400 |
| After | 3.883 | 1.414 |
| After | 4.307 | 2.205 |
| After | 2.818 | 1.691 |
| After | 3.133 | 1.620 |
| After | 9.238 | 4.387 |
| After | 4.803 | 1.536 |
| After | 6.760 | 2.957 |
| After | 3.222 | 1.763 |
| After | 2.957 | 1.680 |
| After | 5.053 | 1.931 |
| After | 8.312 | 1.675 |
| After | 5.650 | 2.889 |
| After | 7.138 | 3.688 |
| After | 12.468 | 7.363 |
| After | 5.004 | 2.756 |

Supplementary Table 3. Top five variables associated with Mitophagy-animal-related DEPs in the canonical variate pair.

| Canonical_Pair | Variable | Loading | Absolute_Loading | Type | Rank |
| --- | --- | --- | --- | --- | --- |
| CV1 | LAD | -0.505083956 | 0.505083956 | Clinical | 1 |
| CV1 | SUVmean | -0.45512145 | 0.45512145 | Clinical | 2 |
| CV1 | AST | 0.389978597 | 0.389978597 | Clinical | 3 |
| CV1 | SUVmax | -0.379860622 | 0.379860622 | Clinical | 4 |
| CV1 | LVPWd | -0.370171417 | 0.370171417 | Clinical | 5 |
| CV1 | OPA1 | 0.856866144 | 0.856866144 | Protein | 1 |
| CV1 | KRAS | -0.701214909 | 0.701214909 | Protein | 2 |
| CV1 | USP15 | 0.23771584 | 0.23771584 | Protein | 3 |
| CV1 | RAB5A | 0.140972152 | 0.140972152 | Protein | 4 |
| CV1 | NA | NA | NA | NA | 5 |

Supplementary Table 4. Top five variables associated with Thermogenesis-related DEPs in the canonical variate pair.

| Canonical_Pair | Variable | Loading | Absolute_Loading | Type | Rank |
| --- | --- | --- | --- | --- | --- |
| CV1 | EF | -0.436254429 | 0.436254429 | Clinical | 1 |
| CV1 | SUVmax | 0.403268597 | 0.403268597 | Clinical | 2 |
| CV1 | SUVmean | 0.396658495 | 0.396658495 | Clinical | 3 |
| CV1 | CK | 0.330346698 | 0.330346698 | Clinical | 4 |
| CV1 | E.e. | -0.268973917 | 0.268973917 | Clinical | 5 |
| CV1 | KRAS | 0.814913277 | 0.814913277 | Protein | 1 |
| CV1 | CPT1A | -0.466236287 | 0.466236287 | Protein | 2 |
| CV1 | ATP5PF | -0.333041109 | 0.333041109 | Protein | 3 |
| CV1 | MAPK14 | -0.258304713 | 0.258304713 | Protein | 4 |
| CV1 | ATP5PB | -0.220520952 | 0.220520952 | Protein | 5 |

Supplementary Table 5. Top five variables associated with Mitochondrion-related DEPs in the canonical variate pair.

| Canonical_Pair | Variable | Loading | Absolute_Loading | Type | Rank |
| --- | --- | --- | --- | --- | --- |
| CV1 | LAD | -0.351216566 | 0.351216566 | Clinical | 1 |
| CV1 | UA | -0.342089698 | 0.342089698 | Clinical | 2 |
| CV1 | MONO. | 0.298016071 | 0.298016071 | Clinical | 3 |
| CV1 | LVIDd | -0.27167538 | 0.27167538 | Clinical | 4 |
| CV1 | EF | -0.262079362 | 0.262079362 | Clinical | 5 |
| CV1 | FAM162A | 0.296300772 | 0.296300772 | Protein | 1 |
| CV1 | NDUFB10 | 0.277743324 | 0.277743324 | Protein | 2 |
| CV1 | ECH1 | 0.275732443 | 0.275732443 | Protein | 3 |
| CV1 | USP15 | 0.234316254 | 0.234316254 | Protein | 4 |
| CV1 | MAPK14 | 0.224321034 | 0.224321034 | Protein | 5 |
| CV2 | WBC | -0.953260821 | 0.953260821 | Clinical | 1 |
| CV2 | NEUT. | -0.939839343 | 0.939839343 | Clinical | 2 |
| CV2 | MONO. | -0.432719012 | 0.432719012 | Clinical | 3 |
| CV2 | IVSd | -0.264251963 | 0.264251963 | Clinical | 4 |
| CV2 | LDH | -0.238303202 | 0.238303202 | Clinical | 5 |
| CV2 | GRK2 | -0.292495075 | 0.292495075 | Protein | 1 |
| CV2 | C1QBP | 0.17317239 | 0.17317239 | Protein | 2 |
| CV2 | FAM162A | 0.171969869 | 0.171969869 | Protein | 3 |
| CV2 | VPS13A | -0.163514052 | 0.163514052 | Protein | 4 |
| CV2 | USP15 | 0.159562816 | 0.159562816 | Protein | 5 |
| CV3 | LVPWd | 0.468307283 | 0.468307283 | Clinical | 1 |
| CV3 | TC | -0.371016173 | 0.371016173 | Clinical | 2 |
| CV3 | LDL | -0.308223767 | 0.308223767 | Clinical | 3 |
| CV3 | IVSd | 0.298894735 | 0.298894735 | Clinical | 4 |
| CV3 | EF | 0.27924039 | 0.27924039 | Clinical | 5 |
| CV3 | C1QBP | 0.375008629 | 0.375008629 | Protein | 1 |
| CV3 | ACOT13 | 0.29035322 | 0.29035322 | Protein | 2 |
| CV3 | FDPS | 0.248172947 | 0.248172947 | Protein | 3 |
| CV3 | OLFM4 | -0.213323989 | 0.213323989 | Protein | 4 |
| CV3 | STOML2 | 0.204132198 | 0.204132198 | Protein | 5 |
| CV4 | LYMPH. | 0.359890774 | 0.359890774 | Clinical | 1 |
| CV4 | LVIDs | -0.31020264 | 0.31020264 | Clinical | 2 |
| CV4 | LDL | -0.291451725 | 0.291451725 | Clinical | 3 |
| CV4 | EF | 0.291329277 | 0.291329277 | Clinical | 4 |
| CV4 | AST | -0.284744735 | 0.284744735 | Clinical | 5 |
| CV4 | SLC25A11 | 0.332538187 | 0.332538187 | Protein | 1 |
| CV4 | PTPN1 | -0.239526976 | 0.239526976 | Protein | 2 |
| CV4 | MCU | -0.238302405 | 0.238302405 | Protein | 3 |
| CV4 | CISD2 | 0.20042912 | 0.20042912 | Protein | 4 |
| CV4 | RAP1GDS1 | -0.181747176 | 0.181747176 | Protein | 5 |
| CV5 | LVIDs | -0.395259962 | 0.395259962 | Clinical | 1 |
| CV5 | LVIDd | -0.393839516 | 0.393839516 | Clinical | 2 |
| CV5 | E.e. | -0.374266197 | 0.374266197 | Clinical | 3 |
| CV5 | CK | -0.28026811 | 0.28026811 | Clinical | 4 |
| CV5 | UA | -0.236973859 | 0.236973859 | Clinical | 5 |
| CV5 | CHCHD3 | -0.227781596 | 0.227781596 | Protein | 1 |
| CV5 | SLC25A11 | -0.177314921 | 0.177314921 | Protein | 2 |
| CV5 | ACAT1 | -0.173123152 | 0.173123152 | Protein | 3 |
| CV5 | STOML2 | -0.161079234 | 0.161079234 | Protein | 4 |
| CV5 | CYRIB | -0.155256954 | 0.155256954 | Protein | 5 |
| CV6 | LYMPH. | -0.425124711 | 0.425124711 | Clinical | 1 |
| CV6 | UA | 0.380563705 | 0.380563705 | Clinical | 2 |
| CV6 | TG | 0.379423671 | 0.379423671 | Clinical | 3 |
| CV6 | LVIDd | 0.250563342 | 0.250563342 | Clinical | 4 |
| CV6 | SUVmean | 0.250246856 | 0.250246856 | Clinical | 5 |
| CV6 | ACOT13 | -0.279935565 | 0.279935565 | Protein | 1 |
| CV6 | ATP5PB | 0.253787284 | 0.253787284 | Protein | 2 |
| CV6 | OLFM4 | -0.253683199 | 0.253683199 | Protein | 3 |
| CV6 | CHCHD3 | 0.235897894 | 0.235897894 | Protein | 4 |
| CV6 | SDHA | 0.229189867 | 0.229189867 | Protein | 5 |
| CV7 | NTproBNP | -0.413663201 | 0.413663201 | Clinical | 1 |
| CV7 | E.e. | -0.383021614 | 0.383021614 | Clinical | 2 |
| CV7 | LAD | -0.366752143 | 0.366752143 | Clinical | 3 |
| CV7 | LDL | 0.34070808 | 0.34070808 | Clinical | 4 |
| CV7 | LVIDs | -0.294740369 | 0.294740369 | Clinical | 5 |
| CV7 | OLFM4 | 0.354970735 | 0.354970735 | Protein | 1 |
| CV7 | CHCHD3 | -0.302366644 | 0.302366644 | Protein | 2 |
| CV7 | FAM162A | -0.286251043 | 0.286251043 | Protein | 3 |
| CV7 | ATP5PF | -0.279041871 | 0.279041871 | Protein | 4 |
| CV7 | STOML2 | -0.252605733 | 0.252605733 | Protein | 5 |
| CV8 | AST | 0.393949615 | 0.393949615 | Clinical | 1 |
| CV8 | LVIDs | -0.350301057 | 0.350301057 | Clinical | 2 |
| CV8 | LVIDd | -0.314337531 | 0.314337531 | Clinical | 3 |
| CV8 | TC | 0.301559226 | 0.301559226 | Clinical | 4 |
| CV8 | IVSd | 0.258804163 | 0.258804163 | Clinical | 5 |
| CV8 | ATP5PB | 0.417798822 | 0.417798822 | Protein | 1 |
| CV8 | MCU | 0.368128821 | 0.368128821 | Protein | 2 |
| CV8 | IVD | 0.342493592 | 0.342493592 | Protein | 3 |
| CV8 | RAP1GDS1 | 0.342492705 | 0.342492705 | Protein | 4 |
| CV8 | PFDN2 | 0.339431301 | 0.339431301 | Protein | 5 |
| CV9 | MONO. | -0.421322099 | 0.421322099 | Clinical | 1 |
| CV9 | LVIDd | 0.409476559 | 0.409476559 | Clinical | 2 |
| CV9 | LAD | 0.397309387 | 0.397309387 | Clinical | 3 |
| CV9 | LVIDs | 0.365811748 | 0.365811748 | Clinical | 4 |
| CV9 | E.e. | 0.356369539 | 0.356369539 | Clinical | 5 |
| CV9 | GRK2 | 0.450845917 | 0.450845917 | Protein | 1 |
| CV9 | CYRIB | -0.305099149 | 0.305099149 | Protein | 2 |
| CV9 | IVD | 0.240280515 | 0.240280515 | Protein | 3 |
| CV9 | USP15 | -0.218147675 | 0.218147675 | Protein | 4 |
| CV9 | SLC25A11 | 0.171095469 | 0.171095469 | Protein | 5 |
| CV10 | LDH | -0.526083272 | 0.526083272 | Clinical | 1 |
| CV10 | EF | 0.4458614 | 0.4458614 | Clinical | 2 |
| CV10 | CK | 0.365950738 | 0.365950738 | Clinical | 3 |
| CV10 | MONO. | -0.339968995 | 0.339968995 | Clinical | 4 |
| CV10 | NTproBNP | -0.325461618 | 0.325461618 | Clinical | 5 |
| CV10 | FDPS | 0.401473696 | 0.401473696 | Protein | 1 |
| CV10 | CNP | 0.312597128 | 0.312597128 | Protein | 2 |
| CV10 | IMMT | 0.276084884 | 0.276084884 | Protein | 3 |
| CV10 | ACAT1 | 0.217491194 | 0.217491194 | Protein | 4 |
| CV10 | CPT1A | 0.209027888 | 0.209027888 | Protein | 5 |
| CV11 | AST | -0.435876271 | 0.435876271 | Clinical | 1 |
| CV11 | CKMB | 0.349683964 | 0.349683964 | Clinical | 2 |
| CV11 | NTproBNP | -0.3101649 | 0.3101649 | Clinical | 3 |
| CV11 | EF | -0.280010934 | 0.280010934 | Clinical | 4 |
| CV11 | TG | -0.249161461 | 0.249161461 | Clinical | 5 |
| CV11 | OLFM4 | 0.445078318 | 0.445078318 | Protein | 1 |
| CV11 | RAP1GDS1 | 0.425018973 | 0.425018973 | Protein | 2 |
| CV11 | CISD2 | 0.216941819 | 0.216941819 | Protein | 3 |
| CV11 | ATP5PF | 0.21653758 | 0.21653758 | Protein | 4 |
| CV11 | ATP5PB | 0.214801834 | 0.214801834 | Protein | 5 |
| CV12 | TG | -0.48056434 | 0.48056434 | Clinical | 1 |
| CV12 | LVPWd | -0.443284605 | 0.443284605 | Clinical | 2 |
| CV12 | NTproBNP | 0.277300833 | 0.277300833 | Clinical | 3 |
| CV12 | HDL | 0.258018538 | 0.258018538 | Clinical | 4 |
| CV12 | CKMB | -0.252290414 | 0.252290414 | Clinical | 5 |
| CV12 | ATP5PF | 0.260796096 | 0.260796096 | Protein | 1 |
| CV12 | ACOT13 | -0.222272418 | 0.222272418 | Protein | 2 |
| CV12 | CPT1A | 0.217165123 | 0.217165123 | Protein | 3 |
| CV12 | PFDN2 | 0.202943161 | 0.202943161 | Protein | 4 |
| CV12 | USP15 | 0.193919867 | 0.193919867 | Protein | 5 |
| CV13 | LDH | -0.429284591 | 0.429284591 | Clinical | 1 |
| CV13 | MONO. | 0.323151881 | 0.323151881 | Clinical | 2 |
| CV13 | LYMPH. | 0.27813552 | 0.27813552 | Clinical | 3 |
| CV13 | LVPWd | -0.268348211 | 0.268348211 | Clinical | 4 |
| CV13 | NTproBNP | -0.224139454 | 0.224139454 | Clinical | 5 |
| CV13 | STOML2 | 0.309487974 | 0.309487974 | Protein | 1 |
| CV13 | SDHA | 0.297127129 | 0.297127129 | Protein | 2 |
| CV13 | USP15 | -0.220245685 | 0.220245685 | Protein | 3 |
| CV13 | CISD2 | -0.208276788 | 0.208276788 | Protein | 4 |
| CV13 | NDUFB10 | 0.184895197 | 0.184895197 | Protein | 5 |
| CV14 | CK | -0.585732439 | 0.585732439 | Clinical | 1 |
| CV14 | E.e. | 0.50756121 | 0.50756121 | Clinical | 2 |
| CV14 | SUVmax | -0.322078125 | 0.322078125 | Clinical | 3 |
| CV14 | SUVmean | -0.288353645 | 0.288353645 | Clinical | 4 |
| CV14 | NTproBNP | 0.263896193 | 0.263896193 | Clinical | 5 |
| CV14 | GRK2 | 0.516167954 | 0.516167954 | Protein | 1 |
| CV14 | VPS13A | -0.408483703 | 0.408483703 | Protein | 2 |
| CV14 | IVD | -0.289209247 | 0.289209247 | Protein | 3 |
| CV14 | OLFM4 | 0.250486114 | 0.250486114 | Protein | 4 |
| CV14 | RAP1GDS1 | -0.239900433 | 0.239900433 | Protein | 5 |
| CV15 | SUVmean | -0.44158393 | 0.44158393 | Clinical | 1 |
| CV15 | IVSd | -0.375027585 | 0.375027585 | Clinical | 2 |
| CV15 | LVPWd | -0.368424619 | 0.368424619 | Clinical | 3 |
| CV15 | SUVmax | -0.352017266 | 0.352017266 | Clinical | 4 |
| CV15 | TG | 0.222554899 | 0.222554899 | Clinical | 5 |
| CV15 | PFDN2 | 0.540895556 | 0.540895556 | Protein | 1 |
| CV15 | CPT1A | 0.493295197 | 0.493295197 | Protein | 2 |
| CV15 | IVD | 0.488418884 | 0.488418884 | Protein | 3 |
| CV15 | PTPN1 | 0.447643195 | 0.447643195 | Protein | 4 |
| CV15 | USP15 | 0.442928055 | 0.442928055 | Protein | 5 |
| CV16 | CKMB | -0.536059764 | 0.536059764 | Clinical | 1 |
| CV16 | UA | 0.52392273 | 0.52392273 | Clinical | 2 |
| CV16 | LDH | -0.406632615 | 0.406632615 | Clinical | 3 |
| CV16 | CK | -0.338743769 | 0.338743769 | Clinical | 4 |
| CV16 | NTproBNP | -0.287457602 | 0.287457602 | Clinical | 5 |
| CV16 | CHCHD3 | 0.265536358 | 0.265536358 | Protein | 1 |
| CV16 | FAM162A | 0.253923274 | 0.253923274 | Protein | 2 |
| CV16 | CYRIB | 0.244261066 | 0.244261066 | Protein | 3 |
| CV16 | CISD2 | -0.243282263 | 0.243282263 | Protein | 4 |
| CV16 | ATP5PF | 0.229410653 | 0.229410653 | Protein | 5 |
| CV17 | LAD | 0.518375358 | 0.518375358 | Clinical | 1 |
| CV17 | AST | -0.449796131 | 0.449796131 | Clinical | 2 |
| CV17 | CKMB | -0.361976955 | 0.361976955 | Clinical | 3 |
| CV17 | LDH | -0.322928074 | 0.322928074 | Clinical | 4 |
| CV17 | LDL | 0.268228362 | 0.268228362 | Clinical | 5 |
| CV17 | OPA1 | -0.672170719 | 0.672170719 | Protein | 1 |
| CV17 | CNP | -0.367845493 | 0.367845493 | Protein | 2 |
| CV17 | CYRIB | -0.245371804 | 0.245371804 | Protein | 3 |
| CV17 | IVD | -0.204629456 | 0.204629456 | Protein | 4 |
| CV17 | FAM162A | -0.201388312 | 0.201388312 | Protein | 5 |
| CV18 | CKMB | 0.4092309 | 0.4092309 | Clinical | 1 |
| CV18 | SUVmean | 0.302395816 | 0.302395816 | Clinical | 2 |
| CV18 | IVSd | 0.233428701 | 0.233428701 | Clinical | 3 |
| CV18 | SUVmax | 0.200963613 | 0.200963613 | Clinical | 4 |
| CV18 | LVIDd | 0.185374362 | 0.185374362 | Clinical | 5 |
| CV18 | MAPK14 | -0.250561042 | 0.250561042 | Protein | 1 |
| CV18 | OPA1 | -0.246738268 | 0.246738268 | Protein | 2 |
| CV18 | NDUFB10 | -0.218947237 | 0.218947237 | Protein | 3 |
| CV18 | ATP5PF | -0.186648405 | 0.186648405 | Protein | 4 |
| CV18 | ECH1 | -0.1857667 | 0.1857667 | Protein | 5 |
| CV19 | HDL | 0.635403062 | 0.635403062 | Clinical | 1 |
| CV19 | TC | 0.373580559 | 0.373580559 | Clinical | 2 |
| CV19 | SUVmean | 0.361557873 | 0.361557873 | Clinical | 3 |
| CV19 | SUVmax | 0.316511741 | 0.316511741 | Clinical | 4 |
| CV19 | EF | -0.288251304 | 0.288251304 | Clinical | 5 |
| CV19 | ACAT1 | -0.546697052 | 0.546697052 | Protein | 1 |
| CV19 | CHCHD3 | -0.466581321 | 0.466581321 | Protein | 2 |
| CV19 | CNP | -0.437252971 | 0.437252971 | Protein | 3 |
| CV19 | VPS13A | -0.39912212 | 0.39912212 | Protein | 4 |
| CV19 | ECH1 | -0.358540977 | 0.358540977 | Protein | 5 |


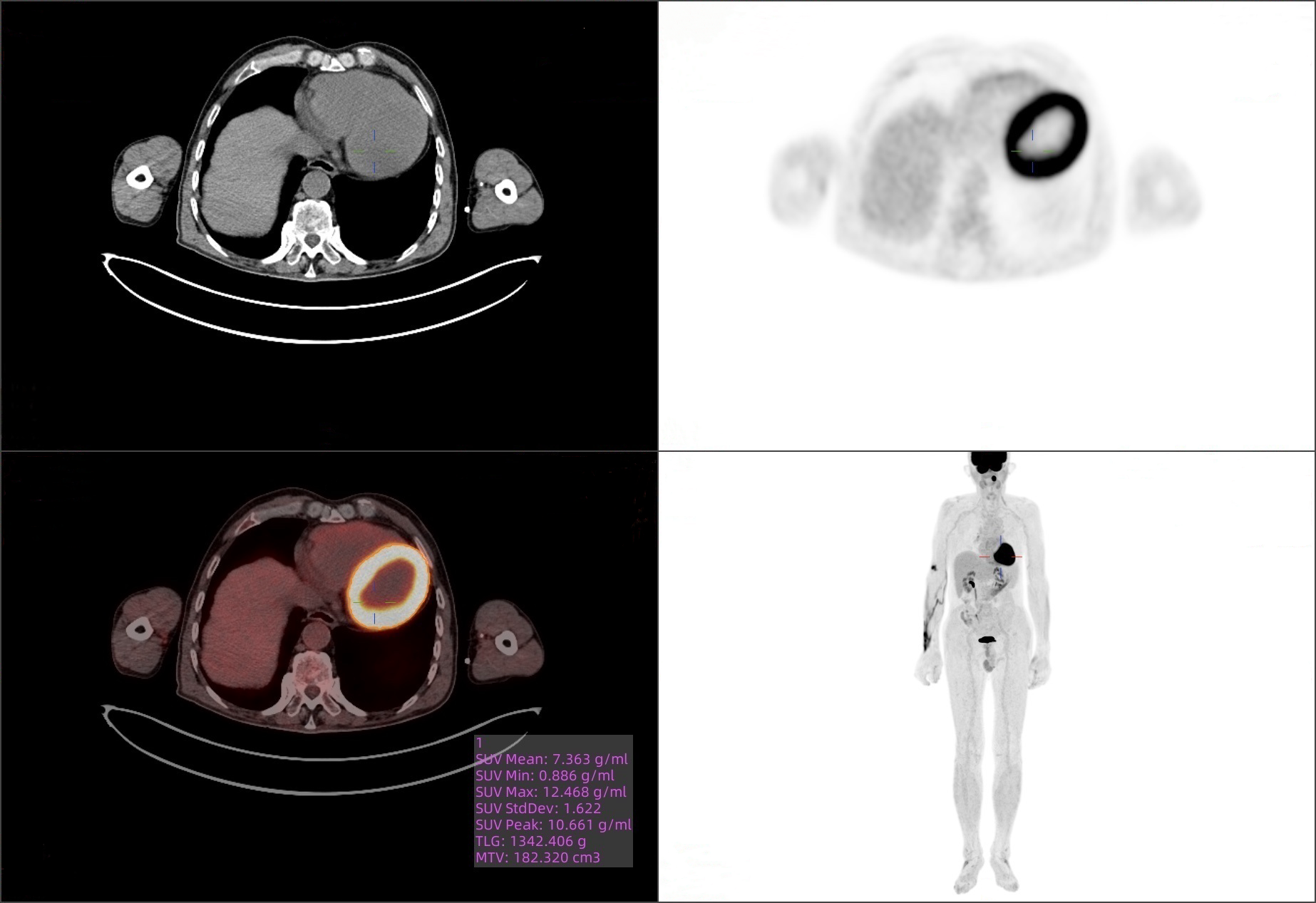


Supplementary Figure 1. Representative ^18^F-FDG PET/CT image demonstrating left ventricular ROI delineation and SUV quantification in an anthracycline-treated patient.


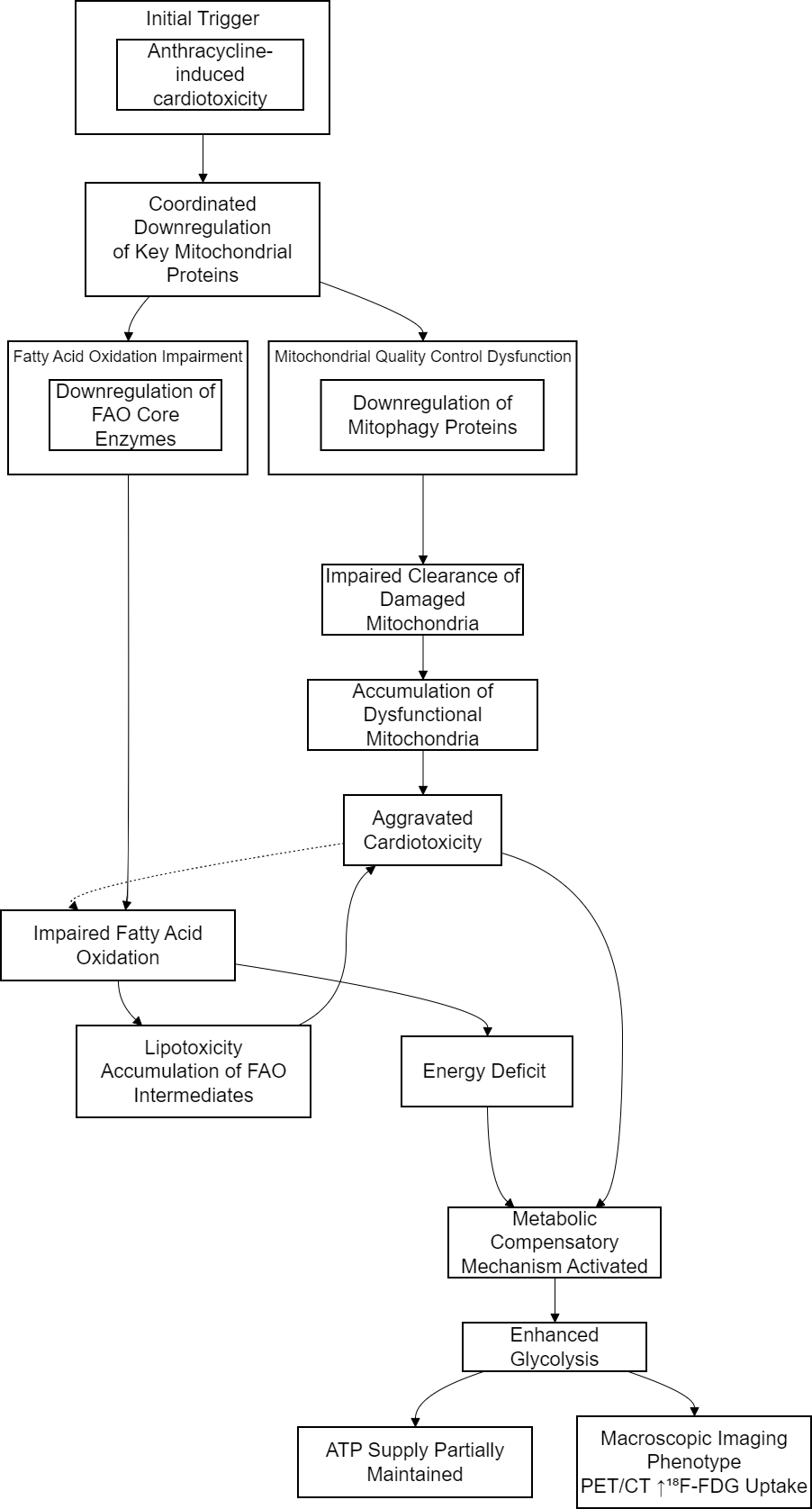


Supplementary Figure 2. Schematic diagram of molecular and imaging integration in myocardial energy metabolism imbalance.
